# Machine learning prediction of post-traumatic osteoarthritis based on three-dimensional printing-derived joint congruence biomechanics in ankle fractures

**DOI:** 10.3389/fmed.2026.1810388

**Published:** 2026-07-23

**Authors:** Xichun Wang, Bin Hu, Wenjie Chen, Hongzhi Yang, Yi Cheng, Kunqiang Chen

**Affiliations:** 1 Department of Orthopedics, Jiujiang No.1 People’s Hospital, Jiujiang, Jiangxi, China; 2 Jiujiang City Key Laboratory of Cell Therapy, Jiujiang, Jiangxi, China; 3 Department of Oncology, Ningguo People’s Hospital, Ningguo, Anhui, China

**Keywords:** ankle fracture, finite element analysis, joint congruence, machine learning, post-traumatic osteoarthritis, predictive model, three-dimensional printing

## Abstract

**Objective:**

Post-traumatic osteoarthritis (PTOA) develops in 20–40% of patients following ankle fracture fixation despite anatomic reduction. This study aimed to develop a machine learning model incorporating three-dimensional (3D) printing-derived joint congruence biomechanics for individualized PTOA prediction.

**Methods:**

This retrospective cohort study included 263 patients (January 2020–January 2024) who underwent preoperative 3D-printed model-assisted surgical planning with minimum 24-month follow-up. Finite element analysis quantified joint congruence parameters including peak contact pressure, pressure inhomogeneity index, and contact center offset. Four machine learning algorithms were developed using training data (*n* = 158, 60%) and validated temporally (*n* = 105, 40%). The primary outcome was PTOA defined by Kellgren-Lawrence grade ≥ II with clinical symptoms.

**Results:**

PTOA developed in 102 patients (38.8%) during mean 36.4-month follow-up. Patients with PTOA demonstrated significantly higher peak contact pressure (15.2 ± 3.8 vs. 9.6 ± 2.4 MPa, *P* < 0.001) and pressure inhomogeneity index (2.8 ± 0.6 vs. 2.3 ± 0.4, *P* < 0.001). In the validation set, the XGBoost full model achieved an area under the curve (AUC) of 0.83 (95% CI 0.75–0.90), significantly outperforming the restricted model without biomechanical variables (AUC 0.74, 95% CI 0.65–0.82; *P* = 0.008). Among 198 patients with anatomic reduction, the full model maintained superior discrimination (AUC 0.81 vs. 0.68, *P* = 0.01), identifying a high-risk subgroup (peak pressure > 13 MPa) with 58.3% PTOA incidence versus 18.2% in low-pressure patients (*P* < 0.001).

**Conclusion:**

Joint congruence parameters from 3D printing-based finite element analysis significantly improve machine learning prediction of PTOA following ankle fracture, identifying high-risk patients even after anatomic reduction.

## 1 Introduction

Ankle fractures represent one of the most common traumatic conditions encountered in orthopedic practice, with an annual incidence ranging from 100 to over 180 per 100,000 population in developed countries (1, 2). Approximately 40% of these injuries require surgical intervention, making ankle fracture fixation among the most frequently performed orthopedic procedures (3). Despite advances in surgical techniques and implant design, post-traumatic osteoarthritis remains the most serious long-term complication, developing in 20–40% of patients within 10 years of surgery, and up to 50% in complex fracture patterns at 20-year follow-up (4, 5). This substantial disease burden translates into significant healthcare costs, including the need for secondary procedures such as ankle arthrodesis or total ankle replacement (6, 7), and profoundly impacts patient quality of life through chronic pain and functional impairment (8, 9).

The traditional paradigm of ankle fracture management centers on anatomic reduction as the primary therapeutic goal, grounded in the assumption that restoration of normal joint geometry ensures favorable long-term outcomes (9). Postoperative assessment relies predominantly on plain radiography, evaluating parameters such as articular step-off, medial and lateral malleolar displacement, and tibiofibular clear space width (10). However, accumulating clinical evidence challenges this conventional approach: even when radiographic criteria for anatomic reduction are satisfied, 20–30% of patients still progress to post-traumatic osteoarthritis during follow-up (11). Conversely, some patients with mild residual displacement remain asymptomatic over extended periods. This dissociation between morphologic restoration and clinical outcomes suggests that two-dimensional static imaging may inadequately capture the biomechanical determinants of joint survival (12).

Three-dimensional printed (3D-printed) technology provides a transformative solution to this challenge. By constructing physical models from preoperative CT data, surgeons can visualize spatial relationships among fracture fragments and simulate reduction procedures (13). More importantly, the digital foundation of these models enables retrospective biomechanical analysis: finite element modeling based on post-reduction geometry can compute contact pressure distribution, stress concentration, and load transmission patterns that are inaccessible through conventional imaging (14). Since the establishment of standardized three-dimensional printing protocols at our institution in 2019, substantial clinical experience has accumulated, providing the technical foundation for large-scale retrospective investigation.

Machine learning offers powerful tools for integrating multidimensional predictors and identifying non-linear relationships (15). Recent applications in orthopedic prognostication have demonstrated superior performance compared with traditional regression models, particularly for complex imaging features (16). Combining biomechanical parameters from three-dimensional printed models with machine learning classification algorithms holds promise for developing precise risk prediction models for post-traumatic osteoarthritis.

We hypothesized that joint congruence parameters derived from finite element analysis would significantly improve prediction of post-traumatic osteoarthritis compared with conventional variables, and would identify high-risk patients even after anatomically satisfactory reduction. This study aimed to develop and validate a machine learning model using 3D-printed model data to enable individualized risk stratification in ankle fracture management

## 2 Materials and methods

### 2.1 Study design and participants

**FIGURE 1:**
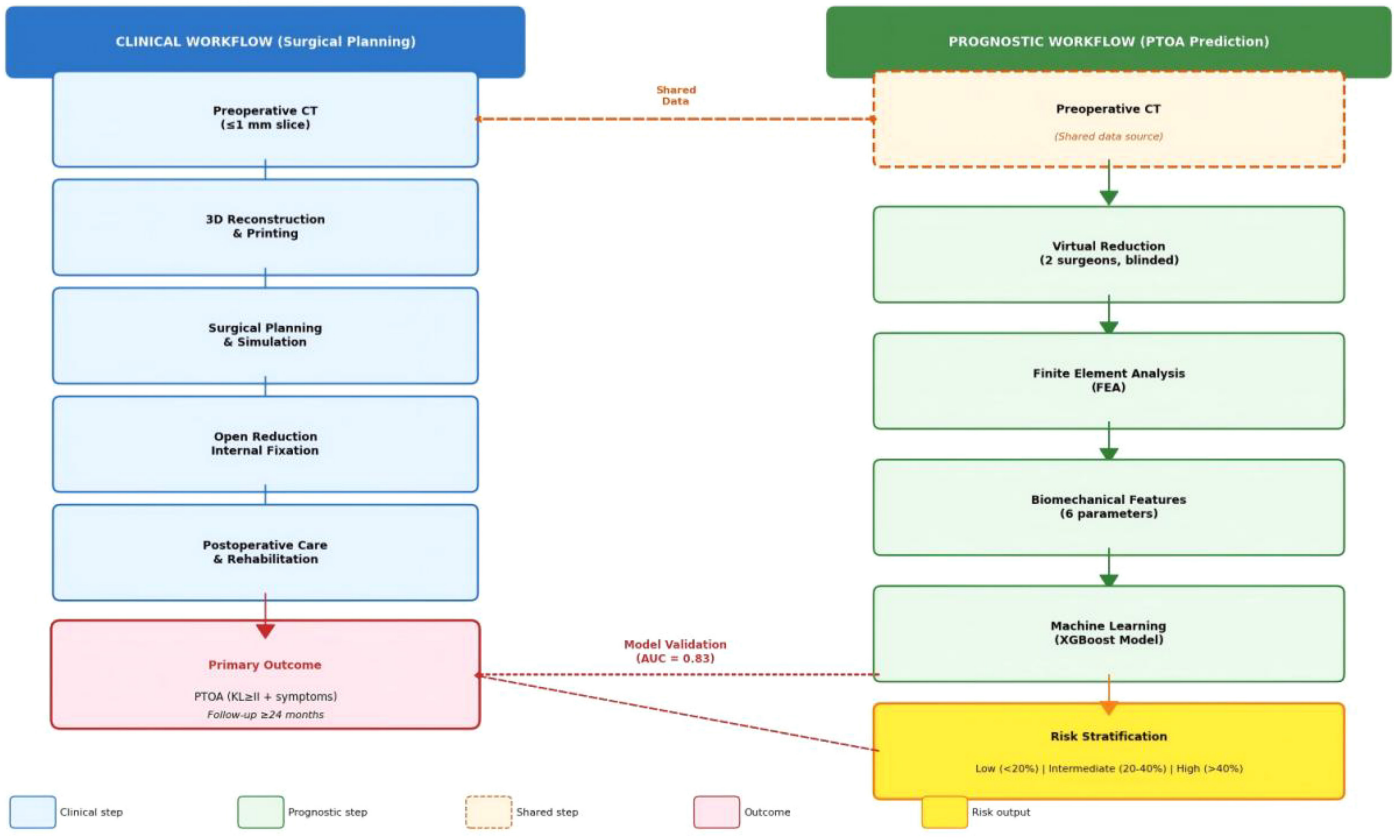
Study framework integrating 3D printing-based surgical planning and prognostic workflow.

The overall study framework is illustrated in Figure 1, which integrates the conventional 3D printing-based surgical planning workflow with the prognostic workflow incorporating virtual reduction, finite element analysis, and machine learning prediction. This single-center retrospective cohort study included consecutive patients with ankle fractures who underwent preoperative 3D-printed model-assisted surgical planning between January 2020 and January 2024. The study protocol was approved by the Institutional Review Board of Jiujiang No.1 People’s Hospital (approval number: JJSDYRMYY-YXLL-2026–050), with waiver of informed consent due to the retrospective design. All procedures followed the Declaration of Helsinki and STROBE guidelines.

Inclusion criteria: (1) unilateral closed ankle fracture treated with open reduction and internal fixation; (2) age 18–75 years; (3) preoperative thin-slice CT (slice thickness ≤ 1 mm) suitable for three-dimensional reconstruction; (4) minimum 24-month postoperative follow-up. Exclusion criteria: open fractures (Gustilo-Anderson type IIIB or IIIC); previous ipsilateral ankle surgery or pre-existing arthritis; inflammatory arthropathy; severe osteoporosis (bone mineral density T-score < −3.0); loss to follow-up or death within 6 months postoperatively; inadequate image quality precluding reliable three-dimensional reconstruction.

Sample size estimation followed guidelines for predictive model development (10–20 events per predictor). Assuming a final model incorporating approximately 15 predictors and an expected post-traumatic osteoarthritis incidence of 40% based on published literature (17), a minimum of 100–130 events would be required, corresponding to a total sample size of 250–325 patients. Accounting for potential missing data and censoring, the target enrollment was set at approximately 260–280 patients.

### 2.2 Three-dimensional printing and model preparation

Preoperative CT imaging was performed using a standardized protocol with a 128-slice multidetector CT scanner. Acquisition parameters included a slice thickness of 0.625 mm, reconstruction interval of 0.5 mm, tube voltage of 120 kVp, and automatic tube current modulation. DICOM datasets were transferred to Mimics Research 25.0 (Materialise, Leuven, Belgium) for segmentation and three-dimensional reconstruction. A semi-automated segmentation approach was employed, combining threshold-based extraction for cortical bone (Hounsfield units 226–3071) with manual refinement to ensure accurate delineation of fracture lines, particularly at the articular surface. The resulting three-dimensional surface models were exported in STL format for virtual manipulation and analysis.

Virtual reduction was performed by two independent orthopedic surgeons using 3D Slicer 5.2.2 and Geomagic Studio 2022 (3D Systems, Rock Hill, SC, United States) in a blinded manner without knowledge of clinical outcomes. Reduction targets were defined according to postoperative radiographic criteria for anatomic reduction: articular step-off less than 1 mm and medial and lateral malleolar displacement less than 2 mm. For patients who underwent early postoperative CT examination (*n* = 79, 30.0% of the cohort), rigid registration algorithms were applied to validate the virtual reduction against actual postoperative anatomy. Agreement was quantified using average surface distance, intraclass correlation coefficient (ICC, two-way random, absolute agreement), and Bland-Altman analysis. An average surface distance less than 1 mm was considered acceptable agreement. Inter-rater reliability was assessed using ICC for surface distance measurements, with discrepancies resolved by a third senior surgeon. Inter-rater reliability was assessed using intraclass correlation coefficients, with discrepancies resolved by a third senior surgeon. Representative models were fabricated using photosensitive resin printing for surgical planning documentation; however, primary analyses utilized virtual models for computational biomechanical assessment.

### 2.3 Finite element analysis

Finite element models were constructed from the post-reduction three-dimensional surface geometry using automated tetrahedral mesh generation. Mesh density was determined through convergence studies, with progressive refinement until changes in peak contact pressure were less than 5%. The final models contained approximately 150,000–250,000 elements in the articular surface region. Material properties were assigned based on published literature: cortical and cancellous bone were modeled as linear elastic isotropic materials with elastic moduli of 17 and 1.5 GPa, respectively, and Poisson’s ratio of 0.30 for both (14, 18). Articular cartilage was represented using a hyperelastic neo-Hookean model with an initial shear modulus of 10 MPa. The cartilage-subchondral bone interface was defined as tied contact, while the tibiotalar joint was modeled as frictionless hard contact to simulate synovial joint lubrication.

Boundary conditions replicated physiological single-leg stance loading. The distal talus was fully constrained to simulate support from the subtalar joint and surrounding ligaments. Axial compression was applied through the tibial shaft at 1.5 times body weight to represent dynamic peak loading during the stance phase of gait. Loading levels were stratified into three categories based on body mass index: low BMI ( < 25 kg/m^2^): 1.3 × body weight; medium BMI (25–30 kg/m^2^): 1.5 × body weight; high BMI ( > 30 kg/m^2^): 1.8 × body weight. This stratification accounts for individual weight-bearing variation while maintaining physiological relevance to dynamic gait loading. Quasi-static equilibrium equations were solved using ABAQUS 2022 (Dassault Systèmes, Vélizy-Villacoublay, France), with convergence verified through residual force norms and contact pressure criteria. Analysis outputs included spatial distribution of contact pressure, von Mises stress, and displacement maps across the tibiotalar joint surface.

### 2.4 Biomechanical and clinical predictors

Candidate biomechanical predictors were extracted from finite element analysis outputs. Peak contact pressure was defined as the maximum nodal pressure value at any location on the articular surface. Mean contact pressure was calculated as the integral of contact pressure over total contact area divided by that area. Pressure inhomogeneity index was computed as the ratio of peak to mean contact pressure, quantifying load distribution uniformity with higher values indicating more pronounced stress concentration. Total contact area represented the surface area bearing non-zero contact pressure. High-pressure area ratio denoted the proportion of contact area with pressure exceeding 10 MPa, a threshold associated with chondrocyte metabolic disturbance in experimental studies. Contact center offset was measured as the Euclidean distance between the geometric center of the talar dome and the pressure-weighted centroid of contact, reflecting articular surface matching. A composite joint congruence index was derived through principal component analysis to integrate individual parameters into a single metric while addressing multicollinearity. For clinical risk stratification, the optimal cutoff value for peak contact pressure was determined using the Youden index on the receiver operating characteristic curve in the training set.

Demographic and clinical variables comprised age at surgery, sex, body mass index, smoking status, diabetes diagnosis, osteoporosis status, and injury energy mechanism (low-energy simple fall versus high-energy trauma including motor vehicle accidents and falls from height). Injury and surgical characteristics included Orthopedic Trauma Association fracture classification, involvement of the posterior malleolus, articular comminution (defined as more than two independent fracture fragments), time from injury to surgery (early less than 24 h, delayed 24–72 h, late more than 72 h), and fixation characteristics including syndesmotic stabilization. Radiographic assessment was performed by a musculoskeletal radiologist blinded to clinical outcomes and three-dimensional analysis. Postoperative anteroposterior and mortise radiographs were evaluated for articular step-off, medial and lateral malleolar displacement, tibiofibular clear space width, and medial clear space width. Anatomic reduction was predefined as articular step-off less than 1 mm and both medial and lateral malleolar displacement less than 2 mm.

### 2.5 Outcome definition

The primary outcome was the development of post-traumatic osteoarthritis, defined using composite criteria to ensure diagnostic specificity. Radiographic criteria required Kellgren-Lawrence grade II or higher on weight-bearing ankle radiographs, indicating definite osteophytes with possible joint space narrowing. Clinical criteria required either a visual analog pain score of 40 mm or greater on a 100 mm scale or an American Orthopedic Foot and Ankle Society ankle-hindfoot score below 80. Both radiographic and clinical criteria had to be satisfied for a positive outcome determination. This composite definition was chosen to maximize diagnostic specificity, as radiographic changes alone may represent incidental age-related findings without clinical significance, whereas clinical symptoms alone may arise from non-osteoarthritic causes such as complex regional pain syndrome or soft tissue dysfunction. Follow-up data were extracted from outpatient clinic records, including serial imaging reports and patient-reported outcome measures. For patients with incomplete follow-up at our institution, structured telephone questionnaires were administered to ascertain functional status and secondary surgeries performed elsewhere. The censoring date was defined as the last clinical contact with adequate imaging and functional assessment.

### 2.6 Machine learning model development

The dataset was divided chronologically into training (January 2020–June 2022, *n* = 158, ∼60%) and validation (July 2022–January 2024, *n* = 105, ∼40%) sets to preserve temporal order and prevent information leakage. This temporal split simulates real-world deployment and provides unbiased estimation of model generalizability.

Missing data were handled using multiple imputation by chained equations for variables with missing rates < 20%, with sensitivity analyses comparing complete case analysis. Categorical variables were one-hot encoded and continuous variables were standardized based on training set distributions. Class imbalance was addressed using synthetic minority over-sampling technique applied only to the training set (PTOA:non-PTOA ratio adjusted to 1:1), while the validation set retained the original distribution to reflect true prevalence.

Four algorithms were evaluated: (1) LASSO-regularized logistic regression (penalty parameters selected via 10-fold cross-validation); (2) Random forest (500 trees, optimized via grid search); (3) XGBoost (tuned using Bayesian optimization for learning rate, depth, and subsampling; optimization targeted area under the precision-recall curve); (4) Cox proportional hazards regression (utilizing complete time-to-event information). Feature importance was assessed via permutation importance (tree-based models) and coefficient magnitude (regularized regression). SHapley Additive exPlanations values were calculated for individualized prediction interpretation.

### 2.7 Statistical analysis and model evaluation

Baseline characteristics were compared between groups using parametric or non-parametric tests for continuous variables and chi-square or Fisher exact tests for categorical variables as appropriate. Survival curves were estimated using the Kaplan-Meier method with log-rank tests for between-group comparisons.

Model discrimination was quantified using the area under the receiver operating characteristic curve for binary classification and time-dependent area under the curve for survival models. Calibration was assessed using the Hosmer-Lemeshow goodness-of-fit test and visual inspection of calibration plots comparing predicted probabilities against observed outcomes. Clinical utility was evaluated through decision curve analysis to estimate net benefit across different threshold probabilities.

The primary comparison tested whether the full model incorporating three-dimensional derived joint congruence parameters significantly outperformed the restricted model containing only demographic, clinical, and conventional radiographic variables. Superiority was defined as a statistically significant increase in area under the receiver operating characteristic curve greater than 0.05 using the DeLong test for correlated curves. Subgroup analyses examined model performance within patients achieving anatomic radiographic reduction and across different fracture types and follow-up durations. Sensitivity analyses evaluated robustness to assumptions regarding missing data mechanisms, alternative outcome definitions, exclusion of virtually reduced cases without CT validation, and restriction to complete follow-up without censoring. Bootstrap resampling with 1,000 iterations provided 95% confidence intervals for performance metrics.

Statistical analyses were performed using Python 3.9.7 with scikit-learn 1.2.0, XGBoost 1.7.0, and lifelines 0.27.0 libraries, and R 4.2.0 with survival 3.3–1 and rms 6.3–0 packages.. Two-sided *P*-values less than 0.05 were considered statistically significant.

## 3 Results

### 3.1 Cohort characteristics

**FIGURE 2:**
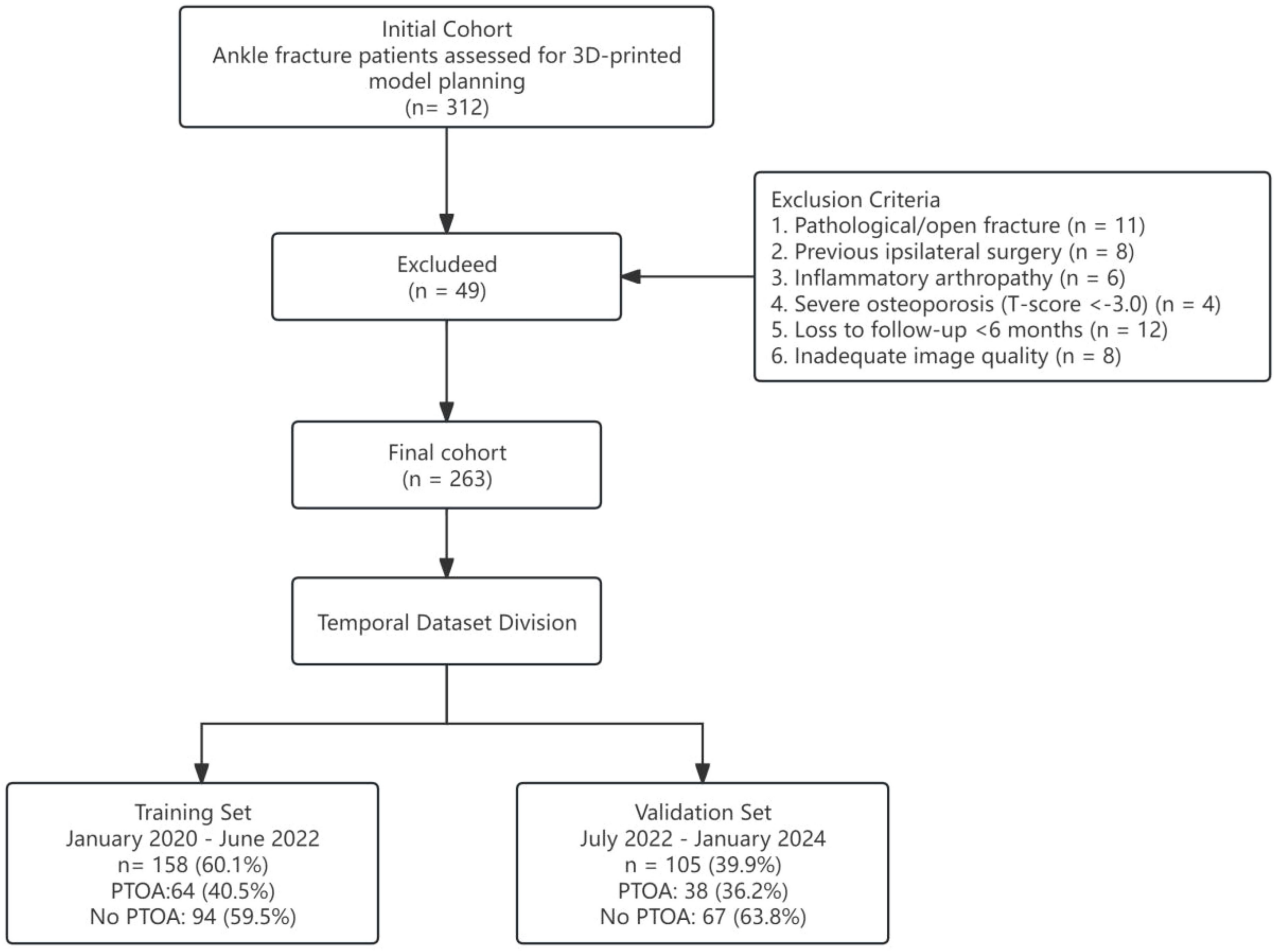
Flowchart of patient selection and study design.

Between January 2020 and January 2024, 312 consecutive patients underwent preoperative 3D-printed model-assisted surgical planning for ankle fractures. After applying exclusion criteria, 263 patients (84.3%) were included in the final analysis, meeting the predetermined sample size requirements (Figure 2). The cohort was chronologically divided into a training set (*n* = 158, January 2020–June 2022) and a validation set (*n* = 105, July 2022–January 2024).

Baseline characteristics were comparable between training and validation sets (all *P* > 0.05, Table 1). The overall cohort had a mean age of 48.3 ± 14.2 years, 54.0% were male, and mean BMI was 26.8 ± 4.2 kg/m^2^. According to OTA/AO classification, 33.8% had type 44A, 48.7% type 44B, and 17.5% type 44C fractures. Posterior malleolar involvement was present in 29.7, and 42.6% had articular comminution. Anatomic reduction was achieved in 75.3% of patients.

**TABLE 1:** Baseline characteristics of the study cohort.

Characteristic	Total (*n* = 263)	Training set (*n* = 158)	Validation set (*n* = 105)	*P*-value
Age, years	48.3 ± 14.2	47.8 ± 14.5	49.1 ± 13.8	0.52
Male sex, n (%)	142 (54.0)	87 (55.1)	55 (52.4)	0.68
BMI, kg/m^2^	26.8 ± 4.2	26.6 ± 4.0	27.1 ± 4.5	0.38
Diabetes, n (%)	54 (20.5)	33 (20.9)	21 (20.0)	0.85
Smoking, n (%)	68 (25.9)	41 (25.9)	27 (25.7)	0.97
High-energy injury, n (%)	85 (32.3)	52 (32.9)	33 (31.4)	0.81
OTA/AO classification, n (%)				0.73
44A	89 (33.8)	52 (32.9)	37 (35.2)	
44B	128 (48.7)	79 (50.0)	49 (46.7)	
44C	46 (17.5)	27 (17.1)	19 (18.1)	
Posterior malleolus involved, n (%)	78 (29.7)	48 (30.4)	30 (28.6)	0.76
Articular comminution, n (%)	112 (42.6)	67 (42.4)	45 (42.9)	0.94
Anatomic reduction achieved, n (%)	198 (75.3)	120 (75.9)	78 (74.3)	0.78
Follow-up duration, months	36.4 ± 8.7	38.2 ± 8.1	33.7 ± 9.0	< 0.001
Post-traumatic osteoarthritis, n (%)	102 (38.8)	64 (40.5)	38 (36.2)	0.51

BMI, body mass index; OTA/AO, Orthopedic Trauma Association/AO Foundation.

Post-traumatic osteoarthritis developed in 102 patients (38.8%) during a mean follow-up of 36.4 ± 8.7 months, with comparable incidence between training (40.5%) and validation sets (36.2%, *P* = 0.51). Patients who developed PTOA were significantly older (52.1 ± 13.8 vs. 45.9 ± 14.1 years, *P* = 0.001), had higher BMI (28.2 ± 4.5 vs. 25.9 ± 3.8 kg/m^2^, *P* < 0.001), and were more likely to have diabetes (28.4% vs. 15.5%, *P* = 0.02) or high-energy injury mechanism (41.2% vs. 26.4%, *P* = 0.02).

### 3.2 Validation of virtual reduction accuracy

**FIGURE 3:**
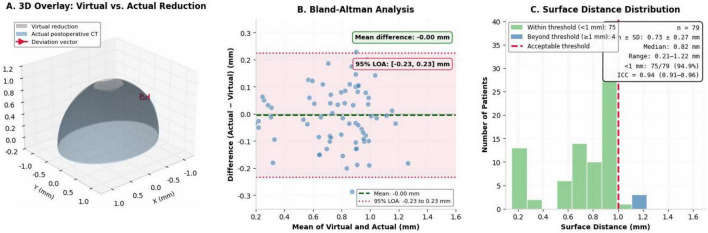
Validation of virtual reduction accuracy against postoperative CT. **(A)** Three-dimensional overlay of virtual reduction (gray) and actual postoperative CT (blue) after rigid registration, with deviation vector (red arrow) indicating surface distance at a representative point. **(B)** Bland-Altman plot comparing virtual and actual surface distances. The mean difference was 0.00 mm, with 95% limits of agreement from - 0.23 to +0.23 mm. **(C)** Distribution of surface distances between virtual and actual reductions. Green bars represent cases within the acceptable threshold ( < 1 mm), and blue bars represent cases beyond the threshold ( ≥ 1 mm). The inset box summarizes key metrics: *n* = 79, mean ± SD 0.73 ± 0.27 mm, median 0.82 mm, range 0.21–1.22 mm, 94.9% (75/79) < 1 mm, ICC 0.94 (95% CI 0.91–0.96).

Among 79 patients (30.0%) with available postoperative CT, the agreement between virtual reduction and actual postoperative anatomy was excellent. The mean surface distance was 0.73 ± 0.27 mm (median 0.82 mm, range 0.21–1.22 mm), with 94.9% (75/79) achieving the prespecified threshold of < 1 mm. Inter-rater reliability was high (ICC = 0.94, 95% CI 0.91–0.96), and Bland-Altman analysis demonstrated minimal systematic bias (mean difference 0.00 mm, 95% limits of agreement −0.23 to +0.23 mm) (Figure 3). Model discrimination was comparable between CT-validated and non-validated subgroups (AUC 0.84 vs. 0.82, *P* = 0.67), supporting the robustness of the virtual reduction approach.

### 3.3 Joint congruence parameters

Finite element analysis revealed significant differences in biomechanical parameters between patients who developed post-traumatic osteoarthritis and those who did not (Table 2 and Supplementary Figure 1). Peak contact pressure was substantially higher in the osteoarthritis group (15.2 ± 3.8 MPa vs. 9.6 ± 2.4 MPa, *P* < 0.001), exceeding the reported threshold of 10 MPa associated with chondrocyte metabolic disturbance. Similarly, mean contact pressure was elevated in the osteoarthritis group (5.8 ± 1.2 MPa vs. 4.2 ± 0.9 MPa, *P* < 0.001). The pressure inhomogeneity index, indicating stress concentration, was significantly greater in patients developing osteoarthritis (2.8 ± 0.6 vs. 2.3 ± 0.4, *P* < 0.001). High-pressure area ratio (contact area with pressure > 10 MPa) was markedly increased in the osteoarthritis group (18.4% ± 8.2% vs. 6.2% ± 4.1%, *P* < 0.001). Contact center offset, reflecting articular surface mismatch, was larger in patients with post-traumatic osteoarthritis (2.8 ± 1.1 mm vs. 1.6 ± 0.8 mm, *P* < 0.001). Total contact area was slightly reduced in the osteoarthritis group (612 ± 98 mm^2^ vs. 658 ± 105 mm^2^, *P* = 0.001). The composite joint congruence index derived from principal component analysis was significantly lower in patients developing osteoarthritis (−0.42 ± 0.85 vs. 0.68 ± 0.72, *P* < 0.001), with lower values indicating worse joint congruence.

**TABLE 2:** Comparison of joint congruence parameters between patients with and without post-traumatic osteoarthritis.

Parameter	PTOA (*n* = 102)	No PTOA (*n* = 161)	*P*-value
Peak contact pressure, MPa	15.2 ± 3.8	9.6 ± 2.4	< 0.001
Mean contact pressure, MPa	5.8 ± 1.2	4.2 ± 0.9	< 0.001
Pressure inhomogeneity index	2.8 ± 0.6	2.3 ± 0.4	< 0.001
Total contact area, mm^2^	612 ± 98	658 ± 105	0.001
High-pressure area ratio ( > 10 MPa), %	18.4 ± 8.2	6.2 ± 4.1	< 0.001
Contact center offset, mm	2.8 ± 1.1	1.6 ± 0.8	< 0.001
Joint congruence index	−0.42 ± 0.85	0.68 ± 0.72	< 0.001

PTOA, post-traumatic osteoarthritis. Data are presented as mean ± standard deviation.

### 3.4 Machine learning model performance

**FIGURE 4:**
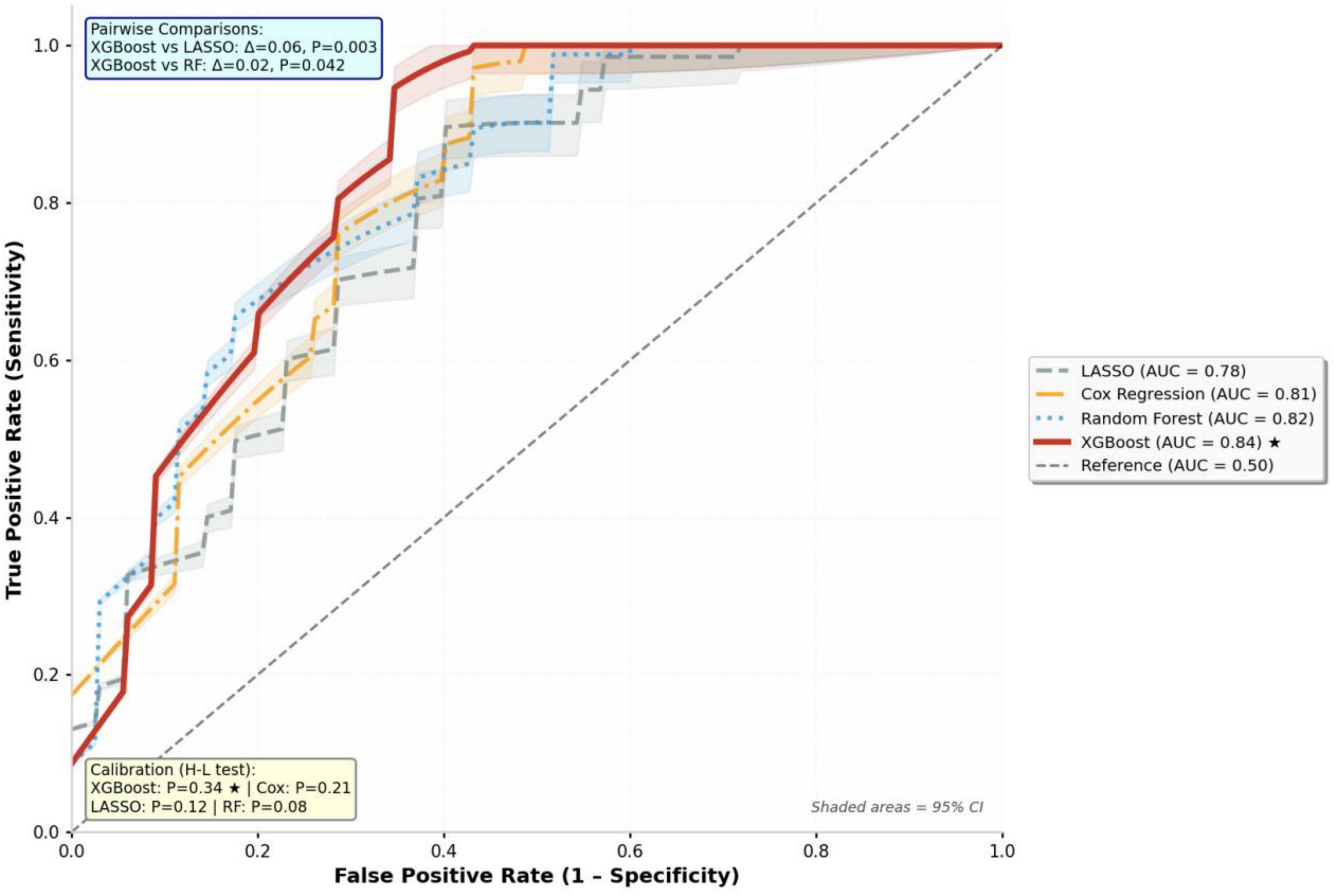
Receiver operating characteristic curves of machine learning models in the training set.

In the training set, all four machine learning algorithms demonstrated acceptable discrimination for post-traumatic osteoarthritis prediction (Figure 4 and Table 3). LASSO-regularized logistic regression achieved an area under the receiver operating characteristic curve of 0.78 [95% confidence interval (CI), 0.71–0.84], while random forest and XGBoost achieved AUCs of 0.82 (95% CI, 0.76–0.88) and 0.84 (95% CI, 0.78–0.89), respectively. Cox proportional hazards regression yielded a time-dependent AUC of 0.81 (95% CI, 0.75–0.87) at 24 months. XGBoost demonstrated the best calibration with a Hosmer-Lemeshow goodness-of-fit *P*-value of 0.34, compared to 0.12 for LASSO, 0.08 for random forest, and 0.21 for Cox regression.

**TABLE 3:** Performance of machine learning models in the training and validation sets.

Model	Training set AUC (95% CI)	Validation set AUC (95% CI)	Calibration (H-L *P*-value)
LASSO logistic regression	0.78 (0.71–0.84)	0.76 (0.67–0.84)	0.22
Random forest	0.82 (0.76–0.88)	0.79 (0.71–0.87)	0.15
XGBoost (full model)	0.84 (0.78–0.89)	0.83 (0.75–0.90)	0.28
XGBoost (restricted model)	0.75 (0.68–0.81)	0.74 (0.65–0.82)	0.18
Cox proportional hazards	0.81 (0.75–0.87)	0.80 (0.72–0.88)	0.31

AUC, area under the receiver operating characteristic curve; CI, confidence interval; H-L, Hosmer-Lemeshow; LASSO, least absolute shrinkage and selection operator. Restricted model included demographic, clinical, and conventional radiographic variables only. Full model added joint congruence parameters.

In the validation set, the XGBoost model incorporating joint congruence parameters achieved an AUC of 0.83 (95% CI, 0.75–0.90), which was significantly higher than the restricted model containing only demographic, clinical, and conventional radiographic variables (AUC 0.74, 95% CI, 0.65–0.82; *P* = 0.008 for difference) (Supplementary Figure 2). The improvement in AUC of 0.09 exceeded the prespecified superiority threshold of 0.05. The full model showed good calibration with observed versus predicted probabilities closely aligned along the diagonal line (Hosmer-Lemeshow *P* = 0.28) (Supplementary Figure 3). Decision curve analysis demonstrated that the full model provided greater net benefit than the restricted model across threshold probabilities ranging from 15 to 80%, indicating superior clinical utility (Supplementary Figure 4). At a 30% risk threshold, the net benefit was 0.24 for the full model compared to 0.15 for the restricted model. Sensitivity analysis stratifying the validation set by follow-up duration ( < 36 months vs. ≥ 36 months) yielded comparable AUCs (0.82 vs. 0.84, *P* = 0.71), suggesting that the follow-up imbalance did not significantly affect model performance.

**FIGURE 5:**
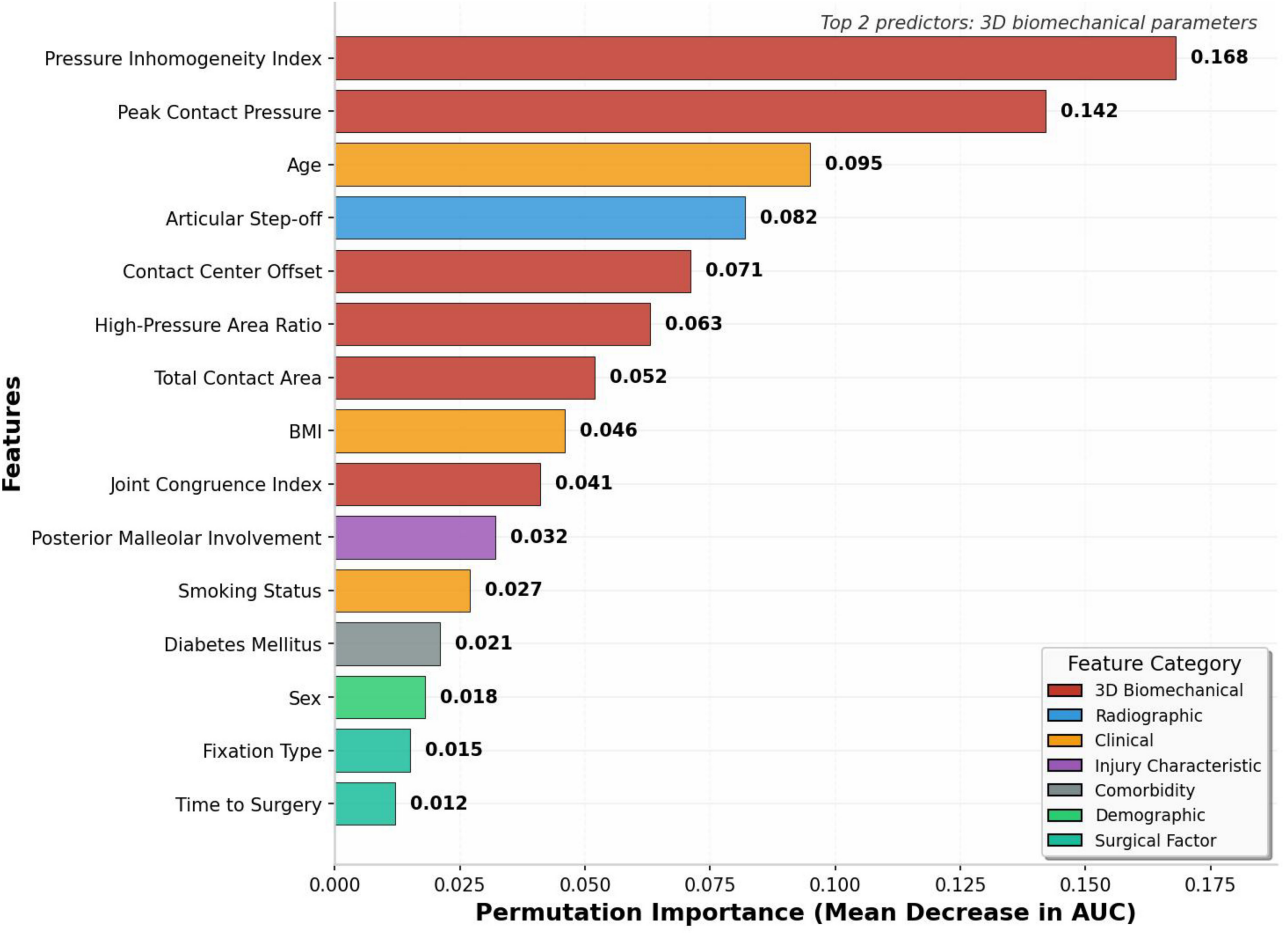
Feature importance ranking in the XGBoost model. Importance is calculated as the mean decrease in Gini impurity for tree-based models.

Feature importance analysis revealed that peak contact pressure ranked highest among all predictors, followed by pressure inhomogeneity index, age, high-pressure area ratio, and body mass index (Figure 5). SHapley Additive exPlanations values indicated that peak contact pressure above 12–13 MPa substantially increased the predicted probability of post-traumatic osteoarthritis, with each 1 MPa increment above this threshold associated with an approximate 8% increase in predicted risk (Supplementary Figure 5).

### 3.5 Subgroup analysis

**FIGURE 6:**
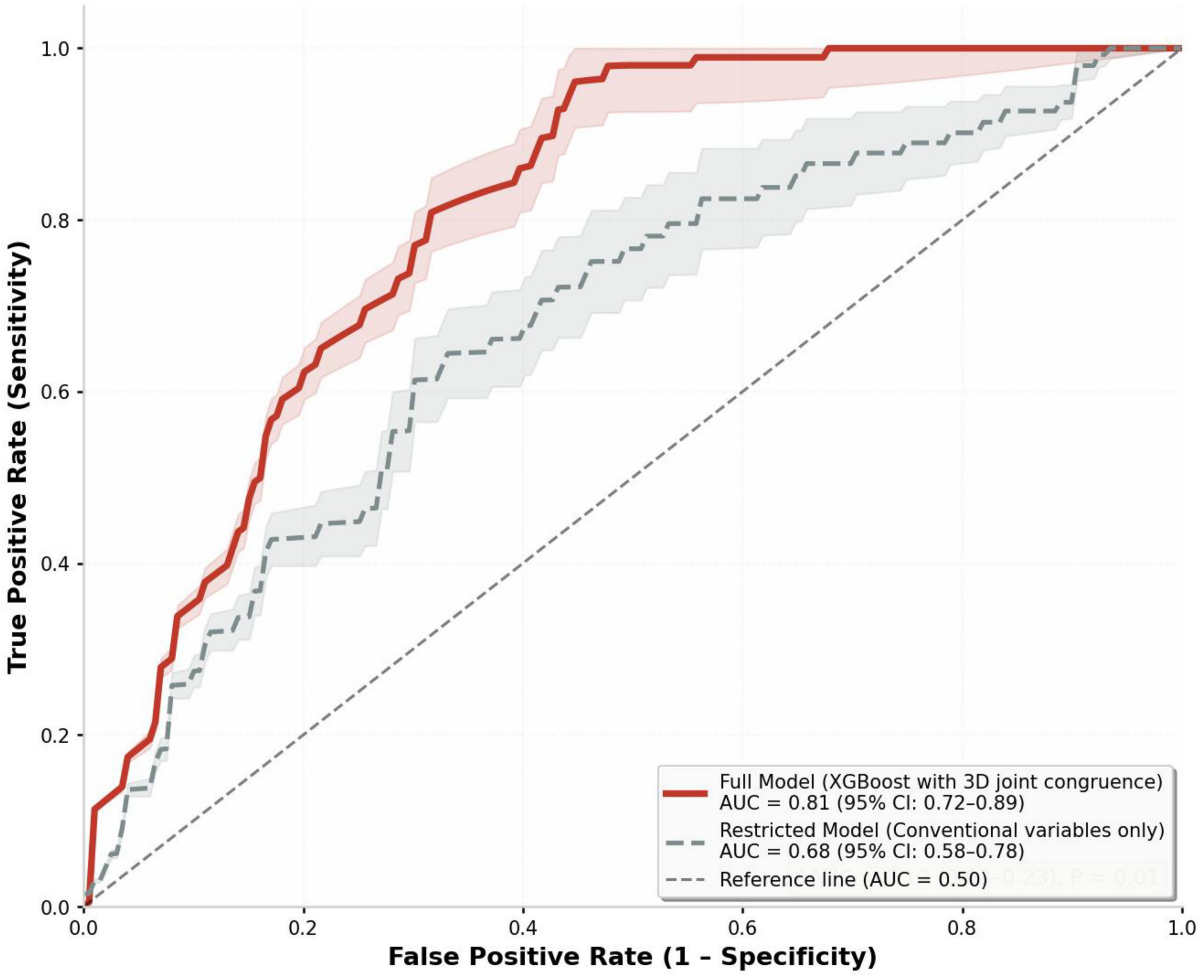
Receiver operating characteristic curves in the subgroup of patients with anatomic radiographic reduction (*n* = 198). The full model incorporating joint congruence parameters significantly outperformed the restricted model (AUC 0.81 versus 0.68, *P* = 0.01).

Among the 198 patients who achieved anatomic radiographic reduction, 68 (34.3%) developed post-traumatic osteoarthritis. In this subgroup, the XGBoost model incorporating joint congruence parameters maintained good predictive performance with an AUC of 0.81 (95% CI, 0.72–0.89), significantly outperforming the restricted model (AUC 0.68, 95% CI, 0.58–0.78; *P* = 0.01) (Figure 6). This finding suggests that biomechanical assessment provides additional prognostic information even when conventional radiographic criteria indicate satisfactory reduction. Based on ROC curve analysis in the training set, the optimal cutoff for peak contact pressure was determined to be 13 MPa (sensitivity 72%, specificity 78%, Youden index 0.50). In the validation set, the 13 MPa threshold achieved a sensitivity of 68% (95% CI 55–80%), specificity of 81% (95% CI 71–89%), positive predictive value of 62% (95% CI 49–74%), and negative predictive value of 85% (95% CI 75–92%), with an overall accuracy of 76% (95% CI 67–84%) (Supplementary Table 1). Patients with anatomic reduction but high peak contact pressure ( > 13 MPa) had a 58.3% incidence of post-traumatic osteoarthritis compared to 18.2% in those with low peak contact pressure ( ≤ 13 MPa) (*P* < 0.001) (Supplementary Figure 6).

Analysis by fracture type showed that the predictive performance was consistent across OTA/AO classifications, with AUCs of 0.85 (95% CI, 0.74–0.94) for type 44A, 0.82 (95% CI, 0.71–0.91) for type 44B, and 0.80 (95% CI, 0.65–0.93) for type 44C fractures. The model performed well in both early follow-up ( < 36 months, AUC 0.84, 95% CI, 0.75–0.92) and late follow-up ( ≥ 36 months, AUC 0.82, 95% CI, 0.71–0.91).

### 3.6 Risk stratification and clinical application

Based on predicted probabilities from the XGBoost model, patients were stratified into low-risk ( < 20%), intermediate-risk (20–40%), and high-risk ( > 40%) categories. In the validation set, the observed incidence of post-traumatic osteoarthritis was 12.5% (5 of 40) in the low-risk group, 37.8% (17 of 45) in the intermediate-risk group, and 70.0% (14 of 20) in the high-risk group (*P* for trend < 0.001) (Table 4 and Supplementary Figure 7). Kaplan-Meier analysis demonstrated significantly different survival curves for the three risk groups (log-rank *P* < 0.001), with median time to osteoarthritis diagnosis of 48 months in the high-risk group versus 28 months in the intermediate-risk group and no events in the low-risk group during the follow-up period (Supplementary Figure 8 **).**

**TABLE 4:** Risk stratification and observed outcomes in the validation set.

Risk category	Predicted probability	n	Observed PTOA, n (%)	Time to PTOA, months
Low	< 20%	40	5 (12.5)	Not reached
Intermediate	20–40%	45	17 (37.8)	28 (20–36)
High	> 40%	20	14 (70.0)	18 (12–26)

PTOA, post-traumatic osteoarthritis. Data presented as median (interquartile range) for time to event.

Sensitivity analyses confirmed the robustness of these findings. Using complete case analysis instead of multiple imputation yielded similar AUC estimates (0.82 vs. 0.83). Excluding patients without CT validation of virtual reduction did not substantially alter model performance (AUC 0.84). Alternative outcome definitions using radiographic criteria alone (Kellgren-Lawrence ≥ II) or clinical criteria alone resulted in AUCs of 0.81 and 0.79, respectively, consistent with the primary analysis.

## 4 Discussion

This study demonstrates that biomechanical parameters of joint congruence derived from finite element analysis of three-dimensional printed models significantly improve machine learning prediction of post-traumatic osteoarthritis following ankle fracture. The incorporation of peak contact pressure, pressure inhomogeneity index, and related biomechanical measures increased the area under the receiver operating characteristic curve from 0.74 to 0.83, exceeding the prespecified superiority threshold and indicating meaningful clinical improvement. Notably, even among patients achieving radiographic anatomic reduction, biomechanical assessment identified a high-risk subgroup with 58% osteoarthritis incidence, suggesting that conventional radiographic criteria may be insufficient for prognostication.

Our findings align with and extend previous investigations into the biomechanical basis of post-traumatic osteoarthritis. Experimental studies have established that static hydrostatic pressure exceeding 10 MPa can induce metabolic disturbances in chondrocytes, including altered matrix synthesis and inflammatory responses (19). In the present cohort, patients developing osteoarthritis demonstrated mean peak contact pressures of 15.2 MPa, substantially exceeding this threshold, whereas those without osteoarthritis averaged 9.6 MPa. This divergence supports the biological plausibility of our biomechanical approach. This may explain our reported incidence of 38.8%, which is higher than the 25% (95% CI 18–32%) reported in a recent systematic review of ankle fracture outcomes (4). The discrepancy likely reflects two factors: first, our longer mean follow-up (36.4 months) captures more incident cases than shorter observation periods; second, our composite definition requiring both radiographic and clinical criteria identifies patients with functionally significant disease that radiographic criteria alone might miss. Sensitivity analyses using alternative outcome definitions yielded comparable model performance (radiographic criteria alone: AUC 0.81; clinical criteria alone: AUC 0.79), supporting the robustness of our approach. Recent patient-specific finite element analyses demonstrate that varus deformities increase peak contact pressure by up to 54% compared to healthy ankles, with stress concentrations shifting medially, particularly beneath the medial malleolus (20). These findings align with computational studies by Li et al. (21), who reported that tibial plafond incongruity significantly alters contact stress distribution. Our results extend these observations by suggesting that finite element analysis can detect subtler abnormalities—focal stress concentrations not apparent on conventional radiographs—that predispose to joint degeneration.

The dissociation between anatomic radiographic reduction and clinical outcomes observed in this study challenges the traditional paradigm that morphologic restoration ensures favorable prognosis. Previous long-term follow-up studies have identified risk factors for PTOA including Weber C fracture pattern, associated medial malleolar fracture, fracture-dislocation, and increasing BMI (5). However, these studies relied predominantly on radiographic and clinical variables without quantitative biomechanical assessment. Our data indicate that two patients with identical radiographic measurements may experience markedly different biomechanical environments, with peak contact pressures varying by 5–8 MPa depending on subtle articular surface geometry. This variability likely explains why a substantial proportion of patients with anatomic reduction still progress to PTOA (4), and supports recent evidence that persistent postoperative step-off > 1 mm significantly increases osteoarthritis risk independent of fragment size (22). The integration of biomechanical assessment into clinical workflow could enable risk stratification that guides follow-up intensity and rehabilitation protocols.

Three-dimensional printing technology provides a transformative solution to the limitations of conventional imaging in ankle fracture management. Systematic reviews and meta-analyses have demonstrated that 3D-printed models for preoperative planning significantly reduce operation duration, intraoperative blood loss, and fluoroscopy usage while improving functional outcomes measured by AOFAS scores (13). However, prior applications have focused primarily on surgical simulation and anatomical visualization rather than quantitative biomechanical analysis. Our study extends these applications by leveraging the digital foundation of 3D-printed models for retrospective finite element modeling, enabling computation of contact pressure distribution and stress concentration patterns inaccessible through conventional imaging. This approach aligns with emerging biomechanical guidance systems that utilize intraoperative assessment of articular reduction and joint contact stress to improve reduction accuracy and minimize post-traumatic arthritis risk (23).

The machine learning methodology employed in this study offers several advantages over traditional statistical approaches for orthopedic prognostication. Recent systematic reviews highlight the expanding role of machine learning in predicting osteoarthritis progression, with deep learning models achieving C-indices of 0.80–0.85 for structural disease progression (24). However, current applications predominantly utilize publicly available datasets with limited external validation and insufficient interpretability (25). XGBoost’s ability to capture non-linear interactions and handle mixed data types proved particularly valuable given the complex interplay between biomechanical, demographic, and injury-related factors in our cohort. The consistency of model performance across fracture types and follow-up durations suggests robust generalizability, while SHAP values provided clinically interpretable insights—specifically, that each 1 MPa increment in peak contact pressure above 12 MPa increased predicted risk by approximately 8%. This granular, individualized prediction, supported by recent applications of SHAP analysis in hip osteoarthritis detection using gait analysis (26), contrasts with the binary risk categorization offered by conventional regression models and addresses the “black box” criticism frequently leveled at machine learning in orthopedic applications (27).

The superior performance of joint congruence parameters over conventional radiographic measures has important clinical implications for personalized medicine in ankle fracture management. Current postoperative assessment relies on step-off and displacement measurements that presume uniform joint loading and cannot characterize stress distribution patterns (28). Our findings suggest that finite element analysis-derived parameters could identify candidates for early biological interventions such as matrix-associated autologous chondrocyte implantation or osteochondral allografting before irreversible joint damage occurs (29). This preventive approach is supported by evidence that aberrant mechanical loading triggers chondrocyte dysregulation, inflammatory mediator release, and matrix degradation through mechanotransduction pathways involving ROS release and alarmin signaling (30, 31). Targeting high-risk patients identified by our model for enhanced monitoring and potential biological therapy could represent a paradigm shift from reactive treatment of end-stage arthritis to prevention of PTOA progression.

Several avenues for future investigation emerge from this work. Prospective validation of the prediction model in an independent cohort would establish its clinical utility for guiding treatment decisions. Integration of the biomechanical assessment pipeline into preoperative planning software could enable real-time risk stratification, allowing surgeons to modify fixation strategies or adjunctive procedures based on predicted joint loading patterns. Extension to other intra-articular fractures, such as tibial plateau or distal radius fractures, would test the generalizability of the joint congruence concept. Finally, longitudinal biomechanical assessment incorporating postoperative healing and remodeling may refine risk prediction and identify optimal windows for preventive interventions. A potential concern is that virtual reduction represents idealized rather than actual surgical anatomy. However, postoperative CT validation in 79 patients demonstrated excellent agreement (mean surface distance 0.73 mm, ICC 0.94), with 94.9% of cases within the < 1 mm threshold. Comparable model performance between CT-validated and non-validated subgroups (AUC 0.84 vs. 0.82, *P* = 0.67) further supports that virtual reduction artifacts did not systematically bias predictions. Nevertheless, operative factors—including fixation strategy, syndesmotic positioning, and soft tissue tension—may introduce biomechanical effects not captured by geometry alone.

This study has several limitations. The single-center retrospective design limits generalizability. Our cohort demographics (mean age 48.3 years, 54% male, BMI 26.8 kg/m^2^, 32% high-energy injuries) reflect local trauma epidemiology that may differ from other settings. Anatomical variability—including talar dome morphology, tibial plafond inclination, and body size—may influence FEA-derived contact pressures. Our BMI-stratified loading protocol and patient-specific geometric modeling partially address this heterogeneity, but prospective multicenter validation is warranted. Virtual reduction simulates ideal anatomic restoration rather than individual surgical outcomes, though this approach specifically tests whether fracture geometry predisposes to PTOA independent of operative variables. The finite element model relied on simplifying assumptions with expected effects on contact pressure estimation. Isotropic linear elastic bone properties typically underestimate peak pressure by 5–10% compared to anisotropic models, as anisotropic stiffness redistributes load toward compliant regions. The hyperelastic neo-Hookean cartilage representation neglects viscoelastic time-dependent behavior; at physiological loading rates, viscoelastic effects would increase peak estimates by approximately 10–15% above our quasi-static predictions. Similarly, quasi-static loading at 1.5 × body weight underestimates dynamic gait peaks of 2.5–3.0 × body weight by 20–30% in absolute magnitude. Importantly, these systematic biases affect absolute pressure values but preserve relative patient rankings, supporting the discriminative validity of our risk stratification approach. Finally, mean 36-month follow-up, while covering the peak PTOA incidence period, may not capture cases with delayed onset.

In conclusion, this study establishes that three-dimensional printing-derived joint congruence biomechanics substantially enhance machine learning prediction of post-traumatic osteoarthritis after ankle fracture. The identification of high-risk patients even among those with anatomic radiographic reduction challenges conventional assessment paradigms and supports the integration of biomechanical analysis into clinical decision-making. These findings provide a foundation for personalized medicine approaches in ankle fracture management, with the ultimate goal of preventing rather than simply predicting post-traumatic joint degeneration.

## Data Availability

The original contributions presented in this study are included in the article/Supplementary material, further inquiries can be directed to the corresponding author.
