# Supplementary material for: Machine learning prediction of post-traumatic osteoarthritis based on three-dimensional printing-derived joint congruence biomechanics in ankle fractures
Source: Front Med (Lausanne). 2026 Jul 23;13:1810388. doi: 10.3389/fmed.2026.1810388 (PMC13441786; doi:10.3389/fmed.2026.1810388)
Supplement: Supplementary file 1 [file Table_1.DOCX]

****Supplementary Figure S1. Comparison of joint congruence parameters between patients with and without post-traumatic osteoarthritis.****

****Supplementary Figure S2. Receiver operating characteristic curves comparing full model (with joint congruence parameters) and restricted model (conventional variables only) in the validation set.****

****Supplementary Figure S3. Calibration plot of the XGBoost full model in the validation set.**** Dashed line represents perfect calibration; solid line represents model calibration with 95% confidence intervals (shaded area).

****Supplementary Figure S4. Decision curve analysis comparing clinical utility of full model and restricted model.**** The full model (solid line) demonstrates greater net benefit than the restricted model (dashed line) across threshold probabilities from 15% to 80%. Dotted lines represent strategies of treating all patients (horizontal) and treating no patients (diagonal).

****Supplementary Figure S5. SHapley Additive exPlanations summary plot showing the impact of features on model output.**** Each point represents a patient, with color indicating feature value (red high, blue low) and horizontal position indicating SHapley Additive exPlanations value contribution to predicted probability of post-traumatic osteoarthritis.

****Supplementary Figure S6. Incidence of post-traumatic osteoarthritis in patients with anatomic radiographic reduction stratified by peak contact pressure.**** High peak contact pressure (>13 MPa) identified a high-risk subgroup despite satisfactory radiographic outcomes.

****Supplementary Figure S7. Observed versus predicted incidence of post-traumatic osteoarthritis across risk categories in the validation set.**** Numbers above bars indicate patient count in each category.

****Supplementary Figure S8. Kaplan-Meier survival curves for time to post-traumatic osteoarthritis according to risk category.**** Log-rank P < 0.001 for comparison across groups.

**Supplementary Table S1. Diagnostic performance of the 13 MPa peak contact pressure threshold in the validation cohort (n=105)**

| Metric | Value (95% CI) |
| --- | --- |
| Sensitivity | 68% (55–80%) |
| Specificity | 81% (71–89%) |
| Positive predictive value | 62% (49–74%) |
| Negative predictive value | 85% (75–92%) |
| Accuracy | 76% (67–84%) |
| Youden index | 0.49 |
| True positives, n | 22 |
| False positives, n | 14 |
| True negatives, n | 59 |
| False negatives, n | 10 |
